# Effect of N-acetyl cysteine, rifampicin, and ozone on biofilm formation in pan-resistant *Klebsiella pneumoniae*: an experimental study

**DOI:** 10.1590/1516-3180.2023.0113.R1.29112023

**Published:** 2024-02-23

**Authors:** Gulsah Tuncer, Zerrin Aktas, Seniha Basaran, Atahan Cagatay, Haluk Eraksoy

**Affiliations:** I MD. Physician, Assistant Professor, Department of Infectious Diseases and Clinical Microbiology, Istanbul Faculty of Medicine, Istanbul University, Istanbul, Turkey.; II PhD. Professor, Department Microbiology and Clinical Microbiology, Istanbul Faculty of Medicine, Istanbul University, Istanbul, Turkey.; III MD. Physician, Assistant Professor, Department of Infectious Diseases and Clinical Microbiology, Istanbul Faculty of Medicine, Istanbul University, Istanbul, Turkey.; IV MD. Physician, Professor, Department of Infectious Diseases and Clinical Microbiology, Istanbul Faculty of Medicine, Istanbul University, Istanbul, Turkey.; V MD. Physician, Professor, Department of Infectious Diseases and Clinical Microbiology, Istanbul Faculty of Medicine, Istanbul University, Istanbul, Turkey.

**Keywords:** Klebsiella pneumoniae, Biofilms, Ozone, Laser scanning confocal microscopy, Quantitative demonstration of biofilm formation, Pan-drug resistance

## Abstract

**BACKGROUND:**

To the best of our knowledge, this is the first study to evaluate the effectiveness of specific concentrations of antibiofilm agents, such as N-acetyl cysteine (NAC), rifampicin, and ozone, for the treatment of pan-resistant *Klebsiella pneumoniae* (PRKp).

**OBJECTIVES:**

We evaluated the effectiveness of antibiofilm agents, such as NAC, rifampicin, and ozone, on biofilm formation in PRKp at 2, 6, 24, and 72 h.

**DESIGN AND SETTING:**

This single-center experimental study was conducted on June 15, 2017, and July 15, 2018, at Istanbul Faculty of Medicine, Istanbul University, Turkey.

**METHODS:**

Biofilm formation and the efficacy of these agents on the biofilm layer were demonstrated using colony counting and laser-screened confocal microscopy.

**RESULTS:**

NAC at a final concentration of 2 μg/mL was administered to bacteria that formed biofilms (24 h), and no significant decrease was detected in the bacterial counts of all isolates (all P > 0.05). Rifampicin with a final concentration of 0.1 μg/mL was administered to bacteria that formed biofilm (24 h), and no significant decrease was detected in bacterial count (all P > 0.05). Notably, ozonated water of even 4.78 mg/L concentration for 72 h decreased the bacterial count by ≥ 2 log_10_.

**CONCLUSION:**

Different approaches are needed for treating PRKp isolates. We demonstrate that PRKp isolates can be successfully treated with higher concentrations of ozone.

## INTRODUCTION

Nosocomial infections are major causes of morbidity and mortality. These infections and their agents are becoming increasingly difficult to treat. Current antibiotics against multidrug-resistant microorganisms are inadequate, and there is a critical shortage of new antibiotics.^1^ Notably, biofilm formation has been implicated as one of the antibiotic-resistance mechanisms.^2^ Living within a biofilm provides bacteria with the advantage of protection against nutrient deprivation, dehydration, pH changes, disinfectants, antibiotics, and toxic substances. Bacteria that form biofilms are 100-1000 times more resistant to antibiotics than their free forms.^3^ The mechanisms responsible for the resistance of bacteria within biofilms often include enzymatic inactivation, efflux pumps, and mutations in drug targets. A microorganism that is inherently susceptible to antimicrobial agents can become resistant when it forms a biofilm and can revert to susceptibility once it detaches from the biofilm. Factors such as low penetration of antimicrobials into the biofilm, changes in the microenvironment, formation of resistant phenotypic variants specific to the biofilm, slowed bacterial growth within the biofilm, and presence of persister cells contribute to antibiotic resistance.^4,5^

## OBJECTIVES

In this study, we aimed to identify an effective method for treating pan-resistant *Klebsiella pneumoniae* (PRKp) isolates, an important nosocomial infection agent in our hospital that has become increasingly difficult to treat. Therefore, there is a need for different approaches to treat potent biofilm-forming pan-resistant PRKp isolates. Antibiotics were hypothesized to be effective after administering effective antibiofilm agents such as N-acetylcysteine (NAC), rifampicin, and ozone.

## METHODS

### Identification of the bacteria isolates and antibiotic susceptibility tests

*K. pneumoniae* isolates that formed invasive infections in the samples collected from nine patients aged over 18 years who were hospitalized in the university hospital between June 15, 2017, and July 15, 2018, were identified using current conventional microbiological and biochemical methods. The zone diameter and minimum inhibitory concentration (MIC) values determined by the European Committee on Antimicrobial Susceptibility Testing (EUCAST) for the disk diffusion, gradient, and broth microdilution methods were used to identify the susceptibility of the bacteria to antibiotics. *Pseudomonas aeruginosa* ATCC 27853, *Escherichia coli* ATCC 25922, NCTC *E. coli* 13846 (mcr-1 positive), *Staphylococcus aureus* ATCC 29213, and *Enterococcus faecalis* ATCC 29212 were used as quality control isolates.^6^

Carbapenem resistance was determined by using the “In-house polymerase chain reaction (PCR)” method for *bla*_OXA-48_, *bla*_NDM-1_, *bla*_VIM_, *bla*_IMI_, and *bla*_KPC_ genes isolates defined by the meropenem zone diameter of < 28 mm in the disk diffusion test as recommended by EUCAST.^3,8-10^

### Identification of the antibiotic resistance of bacteria

Among *K. pneumoniae* isolates, bacteria that are resistant to at least one agent in three or higher antimicrobial categories were defined as “multidrug resistant” (MDR). Bacteria that are sensitive to a maximum of two antimicrobial categories but resistant to at least one agent from other categories are defined as “extremely drug-resistant” (XDR). Finally, bacteria that are resistant to all agents in all antimicrobial categories are defined as “pan-drug resistant” (PDR).^11^

### Demonstration of the biofilm formation

Crystal violet stain was used in the microplate to investigate the biofilm-forming capacity of bacteria.^12^ *P. aeruginosa* ATCC 27853 and *E. coli* ATCC 25922 isolates were used as positive control, and 1% glucose-containing tryptic soy broth (TSB) was used as the negative control.^13^ The values above the optical values measured for negative control were evaluated as biofilm-positive, and the values equal to negative control or below negative control were evaluated as biofilm-negative.^14^

### Investigating the efficacy of NAC, rifampicin, and ozone (0.6 *μ*g/mL) on the biofilm layer

Pure cultures of *K. pneumoniae* isolates of 18-24 hours grown in blood agar were cultured in 5 mL TSB tubes to obtain a suspension of McFarland standard turbidity of 0.5. The tubes were incubated for 18-24 hours at 37 °C. The next day, the bacterial suspension was diluted to a ratio of 1:50 in 5 mL tryptic soy broth containing 1% glucose. 200 *μ*L of the sample was aliquoted to each well of a 96-well U-based cell culture microplate. The fluid medium in the wells was emptied and washed with phosphate-buffered saline (PBS) three times after the microplate was incubated at 37 °C for 18-24 hours. The microplates were dried at room temperature. This stage was defined as basal biofilm formation (0 h). After the evaluation of the basal biofilms, 200 *μ*L of TSB containing 1% glucose was added in each well for 2, 6, 24, and 72 h. The medium was incubated for 18-20 hours, and the dividing colonies were counted. The effects of NAC, rifampicin, and ozone on the biofilm layer were evaluated using the 2 log_10_ method, and comparisons were made to the control group.

### The effect of ozone (4.78 *μ*g/mL) on the biofilm layer

To obtain high rates of ozone concentration, as described for NAC and rifampicin, 1 mL was taken from the prepared bacterial suspension and distributed into a 6-cell culture plates, separately for each bacterium. The plates were incubated at 37 °C for 18-24 hours, and the fluid medium in the plates was emptied and washed with PBS three times. This stage was defined as basal biofilm formation (0 h). Ozone solution in distilled water was prepared by holding the oxygen regulator on an ozone device (Longevity Resources, BC, Canada) at 0.12 speed and 40 gammas for 10 minutes. Ozonated water with a final concentration of 4.78 *μ*g/mL was obtained by measuring with the ozone analyzer device, and 1 mL of the solution was distributed into one of the two 6-cell culture plates prepared for each bacterium. The other plate, in which no ozone solution was added, was used as the control. Plates containing the ozone solution were emptied after 10 min. TSB containing 1% glucose was used to make the well volume 1 mL, and biofilm formation was quantified at 2, 6, 24, and 72 h. We evaluated the cell density at the specified times using a colony count when the final concentration of ozonated water was 4.78 *μ*g/mL, similar to the treatments with NAC and rifampicin.

### Demonstrating the efficacy of the antimicrobial agents on the biofilm formation using laser scanning confocal microscopy

Pure cultures of *K. pneumoniae* isolates of 18-24 hours grown in blood agar were cultured in 5 mL TSB to obtain a suspension of McFarland standard turbidity of 0.5. The tubes were incubated for 18-24 hours at 37 °C. The next day, the bacterial suspension was diluted to a ratio of 1:50 in 5 mL of TSB containing 1% glucose. Samples (2 mL) were taken from this bacterial suspension and delivered in 6-cell culture plates which included sterile circular lamellae prepared separately for each bacterium (to include two plates for each bacteria, at 0, 2, 6, and 24 h). The plates were emptied again at the end of the specified times, washed thrice with PBS solution, and subjected to laser scanning confocal microscopy (Leica, TCS SP8 Ted, Leica Microsystems CMS GmbH, Mannheim, Germany, using 10*x* objective and a 1*x* magnification factor) to evaluate the efficacy of the biofilm layer. After a mixture of fluorescein diacetate (FDA)/propidium iodide (PI 25/2.5 *μ*l/ml^-1^ was prepared, the lamellae were stained for microscopic investigation. Biofilms were observed at 2, 6, and 24 h using laser scanning confocal microscopy.

### Statistical analysis

IBM SPSS for Windows, Version 21.0 (SPSS, IBM Corp., Armonk, NY, USA) was used for statistical analysis. Student’s t-test was used for normal distribution, and the Mann-Whitney U test was used for non-parametric distribution of continuous variables. Wilcoxon Marked Rank Test was performed as a “non-parametric alternative of dependent two-sample t-test,” comparing the two means of the same sample to investigate the significance of the difference of the pre-test and post-test scores of the groups. Statistical significance was set at P < 0.05.

### Ethical approval

All the protocols adhered to the ethical guidelines outlined in the Declaration of Helsinki. This study was approved by the Ethics Committee of Istanbul University and the National Research Committee (approval no: 2017-1414, dated December 8, 2017). Considering the retrospective nature of the study, the requirement for informed consent was waived.

## RESULTS

Nine patients were included in the study. The mean age was 57.6 years (range, 20-93 years), and five patients (55.5%) were male. *K. pneumoniae* isolates were obtained from the blood of four patients, the sputum of three patients, the wound area of one patient, and the urine of one patient. Four patients were followed up for bacteremia, three for ventilator-associated pneumonia, one for surgical area infection, and the others for complicated urinary tract infection. The mean length of hospital stay time was 50.4 days (4-80 days). Seven patients (77.7%) died in a mean of nine days (4-21 days) of treatment initiation, although broad-spectrum antibiotics were administered.

### Antibiotic susceptibility tests and biofilm formation

All *K. pneumoniae* isolates were defined as PDR isolates because they were resistant to all agents in Table 1. The gene responsible for carbapenem resistance in all isolates, identified using the PCR method, was OXA-48, and both OXA-48 and NDM-1 were detected in only one strain (no. 12). The evaluation of all *K. pneumoniae* isolates revealed a biofilm-forming ratio of 100%.

**Table 1.** The minimum inhibitory concentration values and genes responsible for carbapeneme resistance in *K. pneumoniae* isolates (n = 9), tested using the Gradient Test and Fluid Micrudilution methods

Antibiotic susceptibility test	Antibiotic	Isolate no								
		4	6	7	8	9	12	13	14	15
**Gradient test**	Meropenem	> 32	> 32	> 32	24	> 32	> 32	16	32	> 32
	Imipenem	32	24	12	8	> 32	> 32	12	8	8
	Ertapenem	> 32	> 32	> 32	> 32	> 32	> 32	24	> 32	> 32
	Amikacine	24	64	64	48	96	> 256	32	48	> 256
	Gentamicin	32	8	12	48	64	> 256	16	16	> 256
	Ciprofloxacine	> 32	> 32	> 32	> 32	> 32	> 32	> 32	> 32	> 32
	Levofloxacine	> 32	> 32	> 32	> 32	> 32	> 32	> 32	> 32	> 32
	Ceftriaxone	> 32	> 32	> 32	> 32	> 32	> 32	> 32	> 32	> 32
	Ceftroline	> 32	> 32	> 32	> 32	> 32	> 32	> 32	> 32	> 32
	Piperacillin-tazobactam	96	64	64	64	64	256	64	128	256
	Cefepime	> 256	> 256	> 256	> 256	> 256	> 256	> 256	> 256	> 256
	Aztreonam	64	96	96	128	128	128	> 256	32	> 256
	Chloramphenicol	64	64	64	128	96	96	64	128	96
	Trimethoprim-sulphamethoxazole	> 32	> 32	> 32	> 32	> 32	> 32	> 32	> 32	> 32
	Rifampicin	> 32	> 32	> 32	> 32	> 32	> 32	> 32	> 32	> 32
**Fluid microdilution**	Tigecycline	2	2	1	2	1	4	1	1	1
	Colistin	4	8	64	64	32	32	4	8	16
**Agar dilution**	Fosfomycin	125	125	256	> 256	> 256	> 256	> 256	256	125

### Quantification of biofilm formation using the colony counting method

The results for all the isolates are shown in Table 2. 2 *μ*g/mL NAC was administered to bacterial biofilms (24 h), and its effects at 2, 6, 24, and 72 h were investigated. No significant decrease was detected in the bacterial count of all isolates compared to their controls (all P > 0.05). Similar to NAC, 0.1 *μ*g/mL rifampicin was administered to bacterial biofilms (24 h), and no significant decrease was detected in the bacterial count at 2, 6, 24, and 72 h compared to the controls (all P > 0.05).

**Table 2.** The logarithmic colony forming units of N-acetyl Cysteine, rifampicin, and ozone-treated basal biofilm layers, calculated using the colony counting method for each isolate at 2 h, 6 h, 24 h, and 72 h compared with their controls

Isolate no	Difference at 2 h	Result	Difference at 6 h	Result	Difference at 24 h	Result	Difference at 72 h	Result	Antibiofilm agent (mg/L)
	log_10_ CFU/mL		log_10_ CFU/mL		log_10_ CFU/mL		log_10_ CFU/mL		
**4/4 C**	0.17	–	0.03	–	1.49	–	0.04	–	NAC (2)
	0.7	–	0.52	–	0.02	–	0.52	–	RIF (0.1)
	0.14	–	0.8	–	0.52	–	0.42	–	Ozone (0.6)
	0.06	–	0.49	–	0.06	–	2.06	+	Ozone (4. 78)
**6/6 C**	1.27	–	0.12	–	0.14	–	0	–	NAC (2)
	1.1	–	0.66	–	0.3	–	0.12	–	RIF (0.1)
	0.05	–	0.06	–	0.11	–	0.03	–	Ozone (0.6)
	0.11	–	0.06	–	1.27	–	2.4	+	Ozone (4. 78)
**7/7 C**	1.85	–	0.6	–	0.24	–	1	–	NAC (2)
	0.05	–	0.21	–	0.12	–	0.43	–	RIF (0.1)
	0.52	–	0.34	–	0.79	–	0.85	–	Ozone (0.6)
	0.34	–	0.1	–	0.92	–	2.07	+	Ozone (4. 78)
**8/8 C**	1.05	–	0.11	–	0.03	–	0.46	–	NAC (2)
	0.06	–	0.2	–	0.43	–	0.31	–	RIF (0.1)
	0.85	–	0.08	–	0.22	–	0.45	–	Ozone (0.6)
	0.28	–	0.21	–	0.17	–	4.08	+	Ozone (4. 78)
**9/9 C**	1.3	–	0.39	–	1.23	–	0.69	–	NAC (2)
	0.86	–	0.06	–	0.07	–	0.46	–	RIF (0.1)
	0.58	–	0.14	–	0.1	–	0	–	Ozone (0.6)
	0.39	–	0.11	–	0.59	–	3.4	+	Ozone (4. 78)
**12/12 C**	0.29	–	1	–	0.41	–	0.71	–	NAC (2)
	0.72	–	0.1	–	0.14	–	0.28	–	RIF (0.1)
	0.27	–	0.12	–	0	–	0.56	–	Ozone (0.6)
	0.05	–	0.45	–	1.07	–	2.15	+	Ozone (4. 78)
**13/13C**	0	–	0.47	–	0.78	–	0.39	–	NAC (2)
	0.48	–	0.59	–	0.86	–	0.31	–	RIF (0.1)
	1.9	–	1.22	–	0.08	–	0.85	–	Ozone (0.6)
	2.1	–	0.81	–	0.05	–	3.16	+	Ozone (4. 78)
**14/14 C**	0.1	–	0.4	–	0.37	–	0.18	–	NAC (2)
	0.88	–	0.87	–	0.52	–	1	–	RIF (0.1)
	0.9	–	1.46	–	0.88	–	0.13	–	Ozone (0.6)
	1.65	–	0.13	–	0.04	–	3.12	+	Ozone (4. 78)
**15/15 C**	0.4	–	0.96	–	0.28	–	0.21	–	NAC (2)
	0.06	–	0.26	–	0.95	–	0.03	–	RIF (0.1)
	0.08	–	0.7	–	0.59	–	0.02	–	Ozone (0.6)
	0.04	–	0.15	–	0.2	–	3.05	+	Ozone (4. 78)

The efficacy of 0.6 *μ*g/mL and 4.78 *μ*g/mL ozonated water was evaluated on the biofilm layer. When ozonated water of 0.6 *μ*g/mL was administered, a statistically significant decrease in bacterial count was observed at 6 h (P < 0.05) and 24 h (P < 0.05) compared to the control group. However, it was concluded to be ineffective as no logarithmic decrease of 2 log_10_ or greater was observed. However, no significant decrease was detected in the bacterial count at 2 and 72 h (all P > 0.05).

Although a statistically significant decrease was detected (all (all P < 0.05) in the bacterial count at 2 and 24 h with 4.78 *μ*g/mL ozonated water, it was considered ineffective, as no decrease over 2 log_10_ was detected. Interestingly, no statistically significant decrease was detected at 6 h at the same concentration, but at 72 h, there was a decrease between 2.06-4.08 (log_10_) in the bacterial count in all isolates, and it was considered effective.

### Demonstrating live and dead/inactive bacteria in the biofilm using laser scanning confocal microscopy

**Figure 1.**
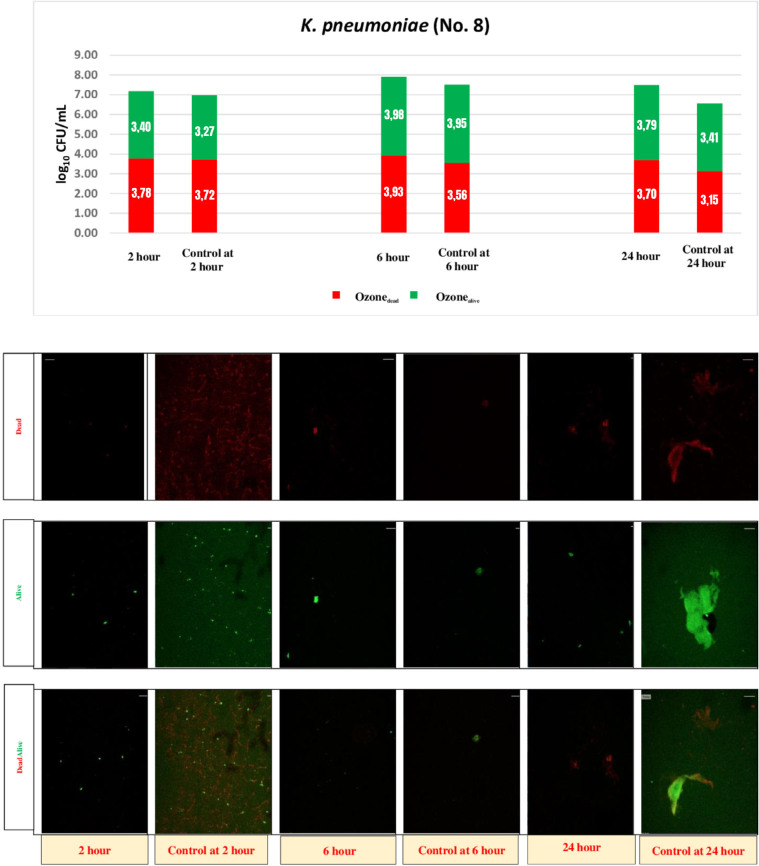
The effects of ozone at 2 h, 6 h, and 24 h on the biofilm of the K. pneumoniae strain no. 8. Cell counts and images of live and dead bacteria in the biofilm of the K. pneumoniae strain no. 8 treated with 4.78 μg/mL ozonated water and the untreated controls calculated using laser scanning confocal microscopy (Red: dead bacteria. Green: alive bacteria).

The results for all isolates are shown in Table 3. The dead and live bacteria 24 h after biofilm formation by strain no. 7 is shown in Figure 1 using the laser scanning confocal microscopy method. The effects on strain 8 at 2, 6, and 24 h after ozonated water treatment (4.78 *μ*g/mL) are shown in Figure 1. Significant decreases were observed on the living (P = 0.05) and death bacterial count (P = 0.01) when using ozonated water at hour 24 h. However, ozonated water did not achieve *≥* 2 log_10_ decrease and was accepted as ineffective.

**Table 3.** The logarithmic cell counts of live and dead cells of each isolate at 2 h, 6 h, and 24 h compared with their controls treated with N-acetyl Cysteine, rifampicin, and ozone, evaluated using the laser scanning confocal microscopy

Isolate n	Difference at h 2	Result	Difference at h 6	Result	Difference at h 24	Result	Antibiofilm agent (mg/L)
	log_10_		log_10_		log_10_		
**4/4C**	0.02 cell/mL	–	0.33 cell/mL	–	0.38 cell/mL	–	NAC^dead^ (2)
	0.87 CFU/mL	–	0.78 CFU/mL	–	0.19 CFU /mL	–	NAC^alive^ (2)
	0.4 cell/mL	–	0.14 cell/mL	–	0.12 cell/mL	–	RIF^dead^ (0.1)
	0.5 CFU/mL	–	0.96 CFU/mL	–	0.02 CFU /mL	–	RIF^alive^ (0.1)
**6/6 C**	0.11 cell/mL	–	0.47 cell/mL	–	0.36 cell/mL	–	NAC^dead^ (2)
	0.14 CFU/mL	–	0.67 CFU/mL	–	0.24 CFU /mL	–	NAC^alive^ (2)
	0.67 cell/mL	–	0.36 cell/mL	–	0.12 cell/mL	–	RIF^dead^ (0.1)
	0.43 CFU/mL	–	0.59 CFU/mL	–	0.4 CFU /mL	–	RIF^alive^ (0.1)
**7/7 C**	0.17 cell/mL	–	0.02 cell/mL	–	0.21 cell/mL	–	Ozone^dead^ (4.78)
	0.03 CFU/mL	–	0.08 CFU/mL	–	0.47 CFU /mL	–	Ozone^alive^ (4.78)
	0.5 cell/mL	–	0.6 cell/mL	–	0.28 cell/mL	–	NAC^dead^ (2)
	0.43 CFU/mL	–	0.08 CFU/mL	–	0.06 CFU /mL	–	NAC^alive^ (2)
	0.48 cell/mL	–	0.63 cell/mL	–	0.23 cell/mL	–	RIF^dead^ (0.1)
	0.22 CFU/mL	–	0.02 CFU/mL	–	0.63 CFU /mL	–	RIF^alive^ (0.1)
**8/8C**	0.19 cell/mL	–	0.02 cell/mL	–	0.54 cell/mL	–	NAC^dead^ (2)
	0.39 CFU /mL	–	0 CFU/mL	–	0.1 CFU /mL	–	NAC^alive^ (2)
	0.06 cell/mL	–	0.37 cell/mL	–	0.55 cell/mL	–	RIF^dead^ (0.1)
	0.13 CFU /mL	–	0.03 CFU/mL	–	0.38 CFU /mL	–	RIF^alive^ (0.1)
**9/9C**	0.12 cell/mL	–	0 cell/mL	–	0.23 cell/mL	–	NAC^dead^ (2)
	0.14 CFU /mL	–	0.01 CFU/mL	–	0.45 CFU /mL	–	NAC^alive^ (2)
	0.4 cell/mL	–	0.44 cell/mL	–	0.24 cell/mL	–	RIF^dead^ (0.1)
	0.14 CFU /mL	–	0.83 CFU/mL	–	0.02 CFU /mL	–	RIF^alive^ (0.1)
**12/12C**	0.53 cell/mL	–	0 cell/mL	–	0.16 cell/mL	–	Ozone^dead^ (4.78)
	0.06 CFU /mL	–	0.09 CFU/mL	–	0.52 CFU /mL	–	Ozone^alive^ (4.78)
	0.68 cell/mL	–	0.23 cell/mL	–	0.33 cell/mL	–	NAC^dead^ (2)
	0.19 CFU /mL	–	0.09 CFU/mL	–	0.3 CFU /mL	–	NAC^alive^ (2)
	0.7 cell/mL	–	0.36 cell/mL	–	0.57 cell/mL	–	RIF^dead^ (0.1)
	0.32 CFU /mL	–	0.11 CFU/mL	–	0.30 CFU /mL	–	RIF^alive^ (0.1)
**13/13 C**	0.73 cell/mL	–	0.41 cell/mL	–	0.52 cell/mL	–	NAC^dead^ (2)
	0.15 CFU /mL	–	0.13 CFU/mL	–	0.33 CFU /mL	–	NAC^alive^ (2)
	0.4 cell/mL	–	0.37 cell/mL	–	0.29 cell/mL	–	RIF^dead^ (0.1)
	0.5 CFU /mL	–	0.3 CFU/mL	–	0.51 CFU /mL	–	RIF^alive^ (0.1)
**14/14 C**	0.03 cell/mL	–	0 cell/mL	–	0.16 cell/mL	–	NAC^dead^ (2)
	0.33 CFU /mL	–	1.19 CFU/mL	–	0.64 CFU /mL	–	NAC^alive^ (2)
	0.27 cell/mL	–	0.79 cell/mL	–	0.39 cell/mL	–	RIF^dead^ (0.1)
	0.45 CFU /mL	–	0.75 CFU/mL	–	0.43 CFU /mL	–	RIF^alive^ (0.1)
**15/15 C**	0.06 cell/mL	–	0.09 cell/mL	–	0.2 cell/mL	–	Ozone^dead^ (4.78)
	0.03 CFU /mL	–	0.15 CFU/mL	–	0.15 CFU /mL	–	Ozone^alive^ (4.78)
	0.82 cell/mL	–	0.41 cell/mL	–	0.64 cell/mL	–	NAC^dead^ (2)
	0.13 CFU /mL	–	0.23 CFU/mL	–	0.45 CFU /mL	–	NAC^alive^ (2)
	0.76 cell/mL	–	0.42 cell/mL	–	0.74 cell/mL	–	RIF^dead^ (0.1)
	0.24 CFU /mL	–	0.71 CFU/mL	–	0.13 CFU/mL	–	RIF^alive^ (0.1)

## DISCUSSION

The biofilm-forming ability of nosocomial opportunistic microorganisms such as *K. pneumoniae* on tissue surfaces is a critical stage in the development of infection. Therefore, it is necessary to obtain detailed information on biofilm formation and biofilm-forming bacteria for the treatment of associated infections.

Copur et al.^15^ showed that the most commonly used meropenem and colistin combination did not affect the planktonic and biofilm forms of *K. pneumoniae* isolates; therefore, meropenem and colistin are not appropriate options for the treatment of *K. pneumoniae-*related infections.

Extracellular polysaccharide (EPS) production increases, particularly at 72 h, as the biofilm becomes older. Diago-Navarro et al.^16^ showed a correlation between the antibiotic resistance profile and biofilm-forming ability by demonstrating the mucoid phenotype of *K. pneumoniae* isolates (n = 40). Singla et al*.* ^17^ reported that the production of the polysaccharide component of the matrix increased in younger biofilms compared to older biofilms, which might be responsible for antibiotic resistance. The results of the same study indicated that an increase in EPS production made the older biofilm resistant to antibiotics, and early initiation of antibiotics to bacteria in the biofilm is more effective.

Various studies have demonstrated that irreversible adhesion to different surfaces, such as catheters and implants, develops within 20 min to 4 hours, ^18-20,^ and biofilm develops within a short time, such as 24 hours.^21,22^

NAC is produced from cysteine residues. NAC has been reported to decrease biofilm formation in various bacteria *in vitro*. In addition, NAC disintegrates biofilm and prevents biofilm formation by reducing EPS production.^23,25^ However, it was found to be ineffective in our study.

Rifampicin has been shown to inhibit the synthesis of bacterial proteins against Gram-positive bacteria.^26^ Furthermore, studies have investigated the efficacy of rifampicin on biofilms formed by Gram-positive bacteria.^27-29^ Rifampicin has recently been used in antibiotic combination studies owing to the difficulties encountered in the treatment of PRKp infections. We found no studies on the efficacy of rifampicin on biofilms of gram-negative bacteria belonging to the *Enterobacteriaceae* family. Therefore, the effects of rifampicin on biofilms were investigated in this study. However, we found it to be ineffective.

Ozone is the trivalent (O_3_) form of oxygen (O_2_) formed in the atmosphere. Some possible explanations for the mechanisms of action are the production of peroxides by ozonolysis, the production or activation of reactive oxygen species, and increased expression of enzymes in cells that have antioxidant activity. The bacteria, fungi, and viruses in the infected tissues are killed by the higher oxygen levels in the tissues, healthy cells reproduce more rapidly, and a stronger immune response is obtained.

Ozone was proven to be useful as an antimicrobial agent in various areas such as medicine, agriculture, maritime, and food sectors.^30,31^ The effects of different concentrations of ozone in dynamic and static cultures, in gas (0.1-20 ppm) and fluid (0.5 ppm), on different bacterial isolates were investigated.^32,34^ In our study, the two different ozone concentrations used in static conditions were 0.6 mg/L for 15 min and 4.78 mg/L for 10 min. The treatment of 4.78 mg/L ozonated water was effective against each bacteria compared to their controls (2.06-4.08 log_10_ decrease) at 72 h. The effect of ozone clearly increased with an increase in ozone concentration. However, in this study, the concentration of 4.78 mg/L was the highest obtainable with the device used for ozonated water. Gürsoy et al.^35^ established in their study on *E. coli* and *S. aureus* that the bacterial count reached zero at 40 min and 3 h, respectively, with an increase in ozone concentration. We suggest that the effect of ozone might be stronger, and more successful results could be obtained if we could obtain higher ozone concentrations (80-100 mg/L, etc.). However, this should be tested in future studies. Previous studies have used medical ozone at a concentration between 1 and 100 *μ*g/mL (0.05–5% O_3_) with a mixture of pure ozone and pure oxygen.^36^

Several studies have reported that the efficacy of ozone varies between new and older biofilms. Bialoszewski et al*.* ^37^ showed in their study that older biofilms at 48-72 hours are more sensitive to the bactericidal effect of ozonated water. Similar to other studies, we found that ozonated water was effective on 72 h old biofilm layer.^37,38^

Although a 2.06-4.08 log_10_ decrease was detected in the bacterial count with ozonated water, complete eradication could not be accomplished in our study. This could be because the ozone concentration was lower, all isolates showed a mucoid phenotype, and the bacterial load in the biofilm was significantly higher. The efficacy of ozone varies according to the bacterial count. Studies have shown that ozone is more effective on biofilms with a lower bacterial load. Gürsoy et al*.* ^35^ showed in their study that bacteria might be completely inhibited, particularly at 1.5 × 10^5^ cfu/mL or lower.

In the present study, we obtained quantitative results of the live bacterial load and count in the biofilm using a culturing method, and the ratio of live to dead cells in the biofilm was determined using laser scanning confocal microscopy. Because both methods have advantages and disadvantages, we recommend a combination of both for such biofilm studies.

This is a preliminary study on *K. pneumoniae*. However, in the near future, we intend to conduct molecular genotyping.

## CONCLUSION

The efficacy of NAC, rifampicin, and ozone for the treatment of PDR isolates of *K*. *pneumoniae* was tested at specific concentrations in the present study. The ozonated water at even 4.78 mg/L concentration was found to produce a *≥* 2 log_10_ decrease in bacterial count in biofilms. Our study is significant in that it suggests that effective clearance is possible at higher concentrations of ozone. The present study lays the foundation for future research as a preliminary study.
